# User Experience of the Co-design Research Approach in eHealth: Activity Analysis With the Course-of-Action Framework

**DOI:** 10.2196/35577

**Published:** 2022-08-09

**Authors:** Melanie Tremblay, Christine Hamel, Anabelle Viau-Guay, Dominique Giroux

**Affiliations:** 1 Department of Teaching and Learning Studies Laval University Québec, QC Canada; 2 VITAM Centre de recherche en santé durable Centre intégré universitaire de santé et de services sociaux de la Capitale-Nationale Laval University Québec, QC Canada; 3 Department of Rehabilitation Laval University Québec, QC Canada; 4 Center of Excellence on Aging Quebec Québec, QC Canada

**Keywords:** co-design, caregivers, activity analysis, course-of-action framework, participant experience, intrinsic description, guidelines, affordances

## Abstract

**Background:**

The cocreation of eHealth solutions with potential users, or co-design, can help make the solution more acceptable. However, the co-design research approach requires substantial investment, and projects are not always fruitful. Researchers have provided guidelines for the co-design approach, but these are either applicable only in specific situations or not supported by empirical data. Ways to optimize the experience of the co-design process from the point of view of the participants are also missing. Scientific literature in the co-design field generally provides an extrinsic description of the experience of participants in co-design projects.

**Objective:**

We addressed this issue by describing a co-design project and focusing on the participants’ experiences looking at what was significant from their point of view.

**Methods:**

We used a qualitative situated cognitive anthropology approach for this study. Data were collected on a co-design research project that aimed to support the help-seeking process of caregivers of functionally dependent older adults. The methodology was based on the perspective of experience by Dewey and used the course-of-action theoretical and methodological framework. Data collection was conducted in 2 phases: observation of participants and recording of sessions and participant self-confrontation interviews using the session recordings. We interviewed 27% (20/74) of the participants. We analyzed the data through nonexclusive emerging categorization of themes using the constant comparative method.

**Results:**

In total, 5 emerging themes were identified. The *perception of extrinsic constraints and the effects of the situation* was central and the most important theme, affecting other themes (*frustrating interactions with others*, *learning together*, *destabilization*, and *getting personal benefits*). Co-occurrences between codes allowed for a visual and narrative understanding of what was significant for the participants during this project. The results highlighted the importance of the role of the research team in preparing and moderating the sessions. They also provided a detailed description of the interactions between participants during the sessions, which is a core aspect of the co-design approach. There were positive and negative aspects of the participants’ experiences during this co-design project. Reflecting on our results, we provided potential affordances to shape the experience of participants in co-design.

**Conclusions:**

Potential users are an essential component of the co-design research approach. Researchers and designers should seek to offer these users a positive and contributory experience to encourage participation in further co-design initiatives. Future research should explore how the proposed affordances influence the success of the intervention.

## Introduction

### Background

The scientific community has shown increasing interest in the co-design approach, especially in the field of eHealth, where people work together to design technological solutions to health-related problems [1-4]. Co-design is a human-centered design methodology used in research-action projects to design a product or service [5]. In the co-design approach, end users (or potential users) participate in knowledge creation and idea generation alongside researchers and designers [6]. By engaging users as experts in their experience with a product or service, co-design can foster social innovations in this rapidly changing world [7].

In total, 3 types of benefits to this approach were identified by Steen et al [8]: to the service being designed (ie, improving the creative process), to the users (ie, better fit and higher satisfaction), and to the organization (ie, more successful innovations and better public relations). A systematic review of user involvement revealed that, out of 87 studies using a co-design approach, 52 reported positive contributions to system design, 12 reported negative contributions, and 23 were uncertain [9]. As the authors stated, the relationship between system success (or usability) and user participation “is neither direct nor binary, and there are various confounding factors that play their role.” As participatory research methods can require a great deal of time, effort, and money, why should we take a co-design approach? Moreover, why are some co-design research projects more fruitful than others? Which factors lead to better results?

Some researchers have proposed guidelines for the co-design process. Noorbergen et al [10] proposed 7 guidelines for co-design in mobile health. These guidelines were based on interviews with co-design method experts (n=8) and mobile health system developers (n=8). The participants were not questioned regarding their experience with the co-design approach. Ostrowski et al [11] proposed 10 co-design guidelines for designing social robots. The proposed guidelines are specific to the co-design of social robots and a long-term study design. Cruickshank et al [12] proposed 8 guidelines for a co-design project that aimed to reimagine a large green space in the heart of the city. These guidelines were proposed by the research team to help designers during the co-design study. They were not documented using empirical data.

What is missing is the point of view of participants who engage in a co-design research project; that is, what is significant for them during a co-design session. Participant experience could provide insights into how this research approach can be more contributory [13]. Scientific literature in the co-design domain generally provides an extrinsic description of the experience of participants in co-design projects [5]. It remains unclear what the participants themselves consider significant in their experience during the co-design sessions.

An in-depth understanding of the experience of participants from their own point of view could help researchers understand why some co-design research projects are more effective than others and further help configure or shape the experience for participants. End users are essential in co-design projects and, therefore, researchers should seek to optimize the experience of the co-design process from the point of view of the participant. Here, experience is considered from the perspective of experience by Dewey [14]: “An experience can be distinguished from other experiences when what is experienced ‘runs its course to fulfilment.’”

### Objectives

Our objective was to describe the experience of potential end users acting as co-designers in a co-design eHealth research project. We wanted to describe the intrinsic experience of participants involved in co-design to provide insight into the cognitive aspects underlying their actions during a co-design research project. We wanted to explore whether and how the experience of co-design from the participant’s perspective can inform researchers about which factors result in more fruitful outcomes.

## Methods

### Co-design Project Studied

In this paper, we present the experience of 20 individuals who engaged in a broad co-design research project that aimed to develop an eHealth tool to make the help‑seeking process easier for caregivers of functionally impaired older adults. The research project protocol included co-design sessions (n=8) and advisory committee (AC) meetings (n=3) of 3 hours each. The AC guided the prototype’s progression and ensured that it conformed to the decisions made during the co-design sessions. Participants in the co-design sessions and AC meetings included 3 categories of potential users of the tool: caregivers of functionally impaired older adults, health and social service professionals, and community workers from the community health network. Co-design sessions were held in 11 administrative regions of Quebec, Canada, from May 2017 to June 2018. Different participants took part in each co-design session as the sessions took place in different regions of Quebec. The AC participants remained largely consistent as those sessions were held in a single location.

During the co-design project, we considered participants as co-designers and positioned ourselves (the research team) in a similar role from a cocreation perspective, as proposed by Sanders and Stappers [6]. The research team included 4 researchers from 3 background domains: 1 (25%) in design (MT) and 2 (50%) in occupational therapy, one of whom was the project lead (DG), as well as 1 (25%) research assistant in anthropology. We also had strong democratic concerns for our participants, wanting to enhance their ability to take part following the social justice perspective work of Sen [15]. Therefore, members of the research team acted as moderators during the sessions while making some design decisions between sessions.

We followed the elements of experience [16] model to structure the entire co-design process. This model proposes a structured process to work on different aspects of the design (eg, interaction design, navigation design, and information design). Sessions were organized to enable work on the different design dimensions (planes) of the model in a linear yet iterative manner, as proposed by Garrett [16]. The participants then coconstructed knowledge and artifacts based on the work of the previous group. For example, the objective of co-design session 5 was to develop the information architecture of the tool. The participants used the *requirements* ideas that were identified during the previous 2 sessions (co-design sessions 3 and 4). The co-design sessions included a variety of activities such as discussion, brainstorming, personas, paper prototyping, and user testing. Activities were selected according to the objective of each session and the progression of the design [16]. Co-design sessions were carried out in both plenary sessions and subgroup workshops, whereas the AC always met in plenary sessions. If all subgroup workshops had the same objective (as was the case for co-design session 5), they each typically included a representative of each category of participants. The participants were always free to choose their subgroup. When the objectives were different between the subgroups, the participant composition was sometimes homogeneous in terms of category. The eHealth tool designed was a high-fidelity prototype of a website. The website had a user-generated content orientation with 2 main objectives: offering and finding resources.

A complete description of the research project protocol has been published previously [17], and the results are presented in 3 papers: one focusing on user needs [18], the second presenting the identification of requirements based on user needs [19], and a third presenting the overall process [20]. Although the previous papers discussed the process and the results for each part, this paper describes the participants’ experience of the process from their point of view.

### Study Design

To understand the experience of the co-design research approach, we needed to combine the action with the appropriation of the action or the consequences for the actor. In our study, we used a situated cognitive anthropology research approach informed by the course-of-action framework, focusing on the actions of the participants in real situations [21-23]. The course-of-action framework, developed in French ergonomics [24], considers human activity in terms of how participants interact with the physical and social environment. The focus is on analyzing and describing the activity by prioritizing intrinsic description (from the actor’s point of view), although extrinsic description (from the researcher’s point of view) is still included. This framework shares epistemological proximity with the co-design approach in that it considers the participants to be the experts in their own experience [25]. This research approach requires phenomena of cognition to be studied in their actual context. This method captures significant parts of the activity from the participant’s point of view, in line with the perspective of experience by Dewey, also pointed out by Laudati and Leleu-Merviel [26]. In our case, the activity encompasses everything that the participants (user-co-designers) would go through during their participation in a co-design research project. The focus remains on the significant parts of their experience from their perspective.

We collected data for this study from the first AC meeting to the end of the project. We were not able to collect data during the first 2 co-design sessions as ethical clearance was obtained after these sessions (June 2017). Our study was not initially planned in the co-design protocol. To collect data for our study, we amended the initial ethical clearance of the co-design project (reference 2016-2017-10 MP).

**Figure 1:**
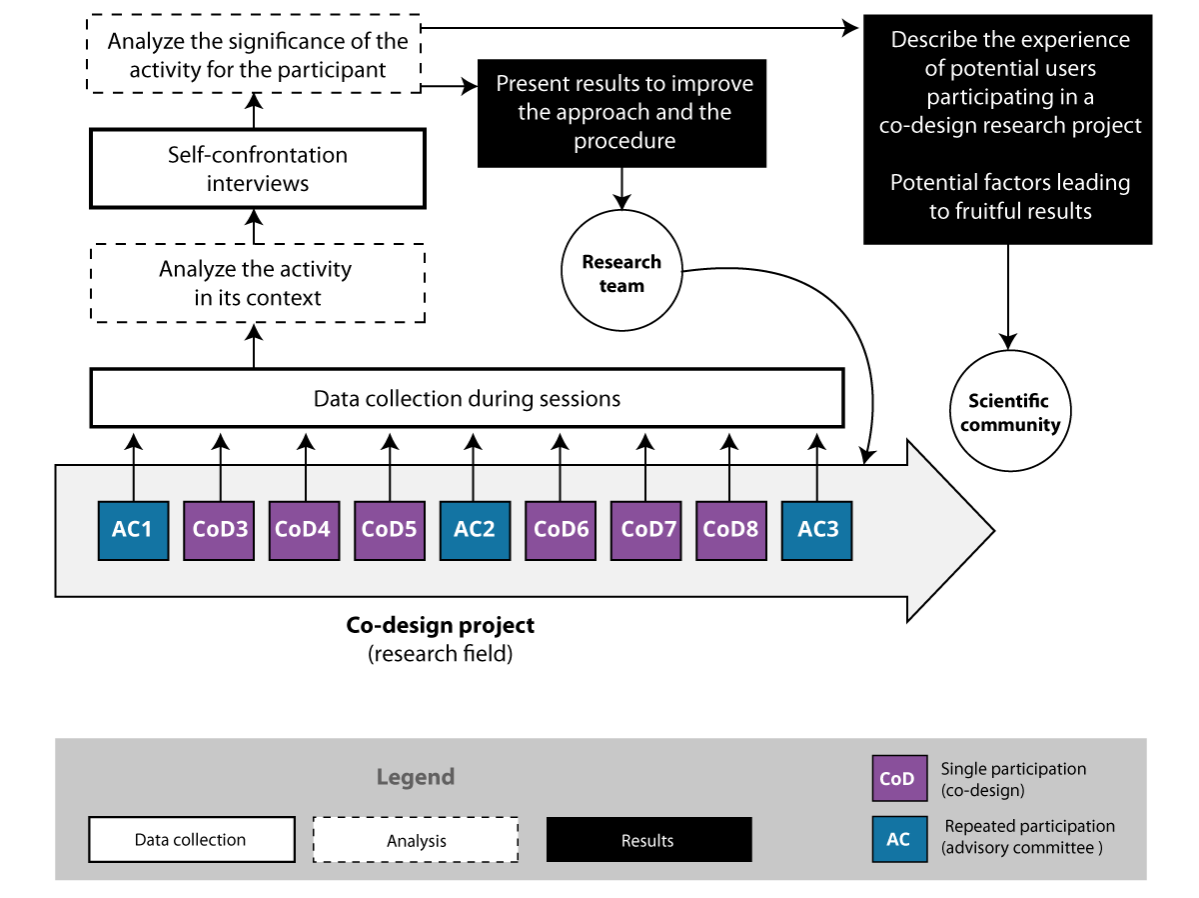
Overview of the study methodology. AC: advisory committee; CoD: co-design session. Adapted from Tremblay [5], which is published under Creative Commons Attribution-NonCommercial-ShareAlike 4.0 International License (CC BY-NC-SA 4.0) [27]).

Important aspects of the intrinsic description of the participants’ experiences were shared with the research team and AC as needed during the debriefing sessions. All data that could not be observed in the sessions and that could inform design decisions and co-design activity planning were discussed after the session with the research team. Important, unclear, or divisive aspects were then negotiated during AC sessions (Figure 1).

### Recruitment

Study participants were recruited at the same time as the participants for the co-design project. They were informed of the opportunity to take part in an individual interview to share their experience of the activity. During the sessions, they could indicate whether they wished to participate in our study. We used purposeful sampling, which is commonly used in qualitative studies [28]. At the end of each session, 2 of the participants who had accepted to take part were selected based on 2 selection criteria (both inclusionary and exclusionary) and contacted for an interview. The selection criteria were as follows: (1) the participant was a potential user of the tool (excluding researchers) and (2) the study achieved a balanced representation of each category of participants (caregivers, health and social service professionals, and community workers). For example, when possible, we selected a health and social service professional instead of a community worker as we had fewer health and social service professionals recruited.

### The 2-Phase Data Collection Process

#### First Phase: Extrinsic Description by the Researchers

As stated by Theureau [22], data should be collected in a 2-phase protocol within the course-of-action research program. The objective of the first phase was to document the extrinsic dimension of the co-design research project (observer description). As previously mentioned, the co-design sessions included plenary and subgroup workshops. During the plenary sessions, a camera and an audio recorder were used. For the subgroup workshop sessions, digital tablets (for video) and audio recorders were used for each group. After the sessions, we used the video recordings to create a summary transcript of the session (the partial chronicle), identifying the principal actor or actors and describing the action for each distinct moment of the session (see the example in Textbox 1).

This partial chronicle was an overview of the session from the researcher’s perspective. It represented all major moments of the session. In this chronicle, we tried to identify what was significant to the participant from our point of view (observer).

**Partial chronicle example with the principal actor and description.**

- Researcher 2 (timestamp 00:00:00): presentation of the project and explanation of the objective of the session. Presentation of the initial user needs that the tool must address. Participants need to find at least two content or functionality requirements for the user need (*know the services offered*).
- Participant 1 and participant 2 (timestamp 00:36:20): participants are talking about the Zarit Grid, a clinical tool used by professionals to evaluate the burden of caregivers and determine which services they are entitled to.

#### Second Phase: Intrinsic Description Provided by the Participants

The objective of the second phase was to document the intrinsic dimension (participant description). During the second phase, we interviewed the participants to obtain a description of the session from their point of view using the recordings of the session and the partial chronicles. Data were collected using a self-confrontation interview protocol [22,29] enriched with facilitation techniques [30].

The self-confrontation method is a semistructured interview that invites participants to describe the activity from their own point of view. On the basis of the identified moment in the partial chronicle, video excerpts of moments of the session were presented to each participant to help them recall their “observable activity that is prereflexive” [31]. Partial chronicles were also used during the interviews to retrieve a specific moment (*t*) of the session, a significant moment shared by the participant but not previously identified by the researcher.

The interviews were structured to facilitate the emergence of the cognitive components of the participants’ course of action. These components are described in the *Data Analysis* section. As stated by Theureau [21], participants will have a natural tendency to describe the given moment using these components. During the interviews, the questions focused on unmentioned components, guiding the participants to verbalize what they were doing, thinking, or feeling at a given moment and what preoccupations and expectations they had.

The transcripts of the self-confrontation interviews (descriptions from the participants) were combined with the partial chronicles (descriptions from the researchers) to arrive at a complete description. Segments of the interviews were replaced with the corresponding moments of the session in the partial chronicle to obtain a complete chronicle. Table 1 summarizes the data collection steps, methods, and instruments.

MT performed all the data collection with guidance from the other authors. MT did not identify herself to the participants as a designer and maintained a passive role as much as possible during the sessions to facilitate data collection. Our hypothesis was that the participants might not be open to sharing their experiences with the designer of the project. MT was identified as a member of the research team.

**Table 1:** Data collection and instruments.

Step	Objective	Method	Instruments
1	Collecting traces of the session—collect data on the session	Observation	Audio and video recording devices (camera, digital tablets, and audio recorders)
2	Partial chronicle—reconstitute the course of events with timestamps	Data condensation of the observation of the session	Audio and video recordings of the session
3	Verbalization—obtain an intrinsic description of the session as experienced by the participant	Self-confrontation interviews	Audio and video recordings of the session Audio and video recording devices (computer and cellular phone) Partial chronicle Interview template
4	Complete chronicle—complete the partial chronicle with the participant’s intrinsic description of the session	Interview transcripts	Audio and video recordings of the session Interview transcript Partial chronicle

### Data Analysis

#### What Constitutes a Sign

The first step in the data analysis within the course-of-action framework is sign reconstitution. On the basis of the theory by Peirce, Theureau [21] proposes the hexadic sign to describe the course-of-action framework, which has 6 components. The hexadic sign demonstrates the cognitive, situated, and dynamic aspects of the activity. Textbox 2 presents the 6 components [31,32] and their definitions. The given moment (*t*) represents a specific moment of the course-of-action framework. It is a recall in the present moment of a series of past structures. The given moment makes it possible to identify a specific moment of the activity corresponding to a given sign. The sign was identified by the researcher (MT) in the transcript of the participants’ verbalization of the session.

**Components of the hexadic sign (interpretation of the studies by Theureau [31] and Ria et al [32]) and their definition.**

- Unit (U): fraction of the activity that could be shown, told, or commented on by the participants at a given moment (*t*). Interpretation, action (practical or communication), emotion, or an area of focus.
- Representamen (R): disruptions (perceptive, mnemonic, or proprioceptive) that are significant to the participant at a given moment (*t*).
- Involvement (E): significant preoccupations and concerns of the participants regarding the representamen (R).
- Expectations (A): expectations at a given moment related to the involvement (E).
- Referential (S): knowledge and experience involved at a given moment (*t*) related to the considered element of the situation (R), the involvement (E), and the expectations (A).
- Interpretant (I): learning or appropriation—confirmation or transformation of the triad (A-E-S) at a given moment (*t*).

**Figure 2:**
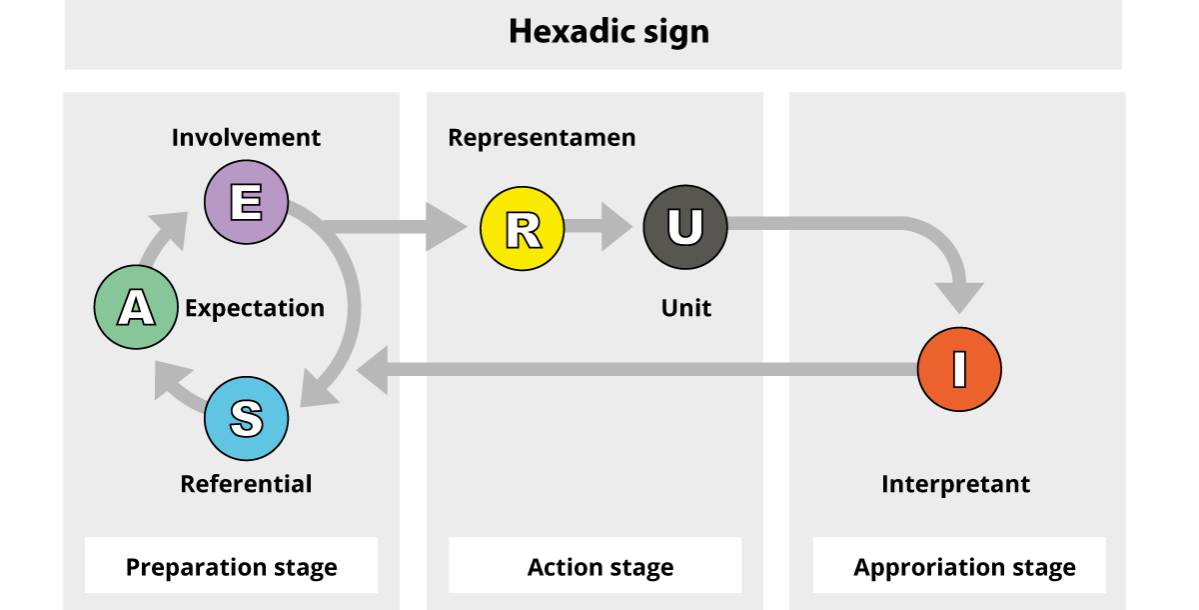
Structural order of components. Adapted from Tremblay [5], which is published under Creative Commons Attribution-NonCommercial-ShareAlike 4.0 International License (CC BY-NC-SA 4.0) [27]).

The components of the hexadic sign are presented in a structured order. As stated by Theureau [21], this order is not temporal but structural. Some components (expectations [A], involvement [E], and referential [S]) reflect the preparation stage (Figure 1). Others (representamen [R] and unit [U]) are related to the perception of the action at a given moment (*t*), and the last component (interpretant [I]) reflects the actor’s appropriation of the experience [33], meaning the validation or invalidation of the A-E-S triad, as shown in Figure 2.

Although the course-of-action framework prioritizes the intrinsic dynamic organization, extrinsic considerations are nevertheless important. The course-of-action framework studies the intrinsic dynamic organization of one or multiple actors and the extrinsic constraints and effects.

The reconstitution of signs was carried out by extracting components (U, R, E, A, S, and I) from the discourse, bringing out the essence of the sign and identifying it. We first identified the unit (U) of the sign from the participants’ comments on what they were doing, thinking, or feeling at a specific moment. To complete the sign, we identified the associated components (R, E, A, S, and I) for that particular unit (U). For example, based on the feeling at this given moment, what were the significant concerns of the participant regarding the element under consideration in the situation (R)? The components were either accompanied by a direct excerpt of the transcript or by a reformulation, attempting to stay as close as possible to what was said by the participant. When we could not directly extract a component from the discourse, we tried to infer it based on the overall experience of the participant. These inferred components were identified (*i*) in the data. This first step of the analysis was performed in table format in Microsoft Word.

#### Emerging Categorization of Signs

After sign identification, the second step of the analysis was to answer the specific research questions. As our objective was to describe the experience of participants from the perspective of experience by Dewey [14], we used an emerging categorization of themes [34] with the constant comparative method [35]. Themes were not mutually exclusive. Signs could be coded with more than one subtheme and, therefore, included in more than one theme. We did not use all the signs in each interview but rather the one or ones that seemed to represent an experience during the session [14]. In total, 2 indicators helped identify these signs along with MT’s own perception of moments that appeared to be an experience for the participants. First, some signs (or moments) were identified during one of the first questions that the participants answered in the self-confrontation interview: *Were there specific moments of the activity that were more important or significant for you?* The participants specified moments of the session, and signs representing those moments were extracted. The second indicator was when several signs were on the same topic. As mentioned by Dewey [14], “[t]here is interest in completing an experience...growing meaning conserved and accumulating toward an end that is felt as accomplishment of a process.” If the participants frequently came back to a specific moment, this moment was more important to them, thereby representing an experience for them. Finally, MT was intensely engaged during all steps of the data collection and analysis process, giving her a deep understanding of the activity of the participants and allowing her to distinguish moments representing an experience for them. Themes emerged from these extracted data.

Intracoder agreement (internal revision) of the themes was performed after 1 month [36]. Subthemes were then identified, helping stabilize the theme categorization. A table of the themes with definitions, subthemes, and 1 example of data for each subtheme was revised by 1 author (CH) to obtain intercoder agreement. The themes and subthemes were negotiated between the researchers and then revised by all the authors, resulting in a slight reformulation to better represent the concept of experience. The coding was revised a third time by the author (MT).

### Ethics Approval

The study received ethical approval from the Comité d'éthique de la recherche sectoriel santé des populations et première ligne (2016-2017-10 MP). Informed consent was obtained from each participant, who also received a nominal compensation of CAD $20 (US $16.45).

## Results

### Participants

From the total number of participants (potential users) in the co-design project (N=74), 20 were recruited for this study. Therefore, we documented the experiences of 27% (20/74) of the participants in the co-design project. We recruited 2 participants for each session except for co-design session 3, where 1 participant withdrew, and co-design session 5, where 6 participants were recruited. Equivalence between the user categories was not completely reached—of the 20 participants, there were 9 (45%) caregivers, 4 (20%) health and social service professionals, and 7 (35%) community workers. A participant in the AC was interviewed twice after the second and third AC sessions. Therefore, the total number of self-confrontation interviews was 21. Table 2 presents the demographic characteristics of the participants included in our study.

**Table 2:** Sociodemographic data of the participants (N=20).

Variable		Caregivers (n=9)	HSSPs^a^ (n=4)	CWs^b^ (n=7)
**Gender, n (%)**				
	Women	8 (40)	4 (20)	4 (20)
	Men	1 (5)	0 (0)	3 (15)
**Age range (years), mean (SD)**				
	25-54	N/A^c^	N/A	43 (9.0)
	31-49	N/A	37 (8.3)	N/A
	44-82	65 (11.6)	N/A	N/A
**Education level, n (%)**				
	Elementary school	0 (0)	0 (0)	0 (0)
	High school	2 (2)	0 (0)	0 (0)
	College	0 (0)	1 (5)	1 (5)
	University	7 (35)	3 (45)	6 (30)

^a^HSSP: health and social service professional.

^b^CW: community worker.

^c^N/A: not applicable.

### Sessions

Table 3 presents the description of the sessions, objectives, and methods used to reach the objective.

**Table 3:** Session description.

Session	Objectives	Methods	Screen capture of the session
Advisory committee 1—Centre-du-Québec	Validate the design decision made during the first 2 co-design sessions on user needs. Address conflicting results.	Plenary discussion. Face-to-face and videoconference participation.	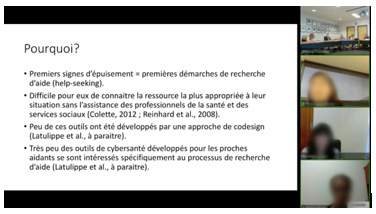
Co-design 3—Saguenay	Identification of user needs already met by other tools and identification of functionalities and content of existing tools related to those needs (what co-designers would keep, modify, or change).	Comparison of existing eHealth information and communication technology tools (websites and apps). Small group workshop using a speed dating approach.	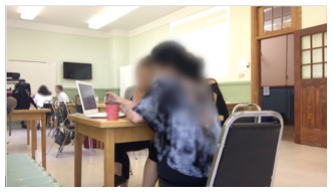
Co-design 4—Bas-Saint-Laurent	Identification of functional or content requirements for the needs not met by existing tools.	Plenary brainstorming and small group workshops.	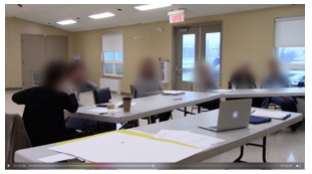
Co-design 5—Gatineau, Outaouais	Prioritization of functional requirements and design of information architecture.	Paper prototyping in small group workshops.	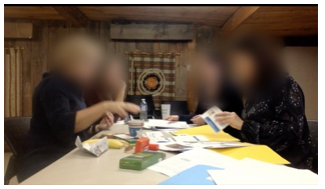
Advisory committee 2—Centre-du-Québec	Decision on conflicting requirements (no consensus reached).	Plenary discussion. A total of 2 documents presenting the results and 3 different clickable PDF prototypes. Face-to-face and videoconference participation.	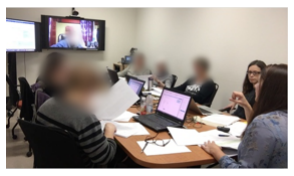
Co-design 6—Montréal-Laval	Information design (content creation).	Plenary presentation and small group brainstorming workshops.	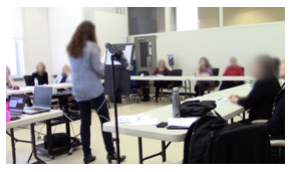
Co-design 7—Trois-Rivières, Mauricie	Information design (content creation).	Plenary presentation and small group brainstorming workshops.	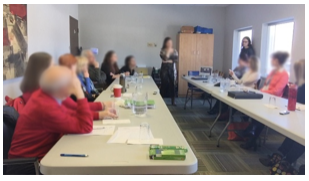
Co-design 8—Montérégie	Information design (content creation) and interface design.	Small group brainstorming workshops. Usability evaluation with a low-fidelity prototype (version 1). Discussion on interface design of the prototype.	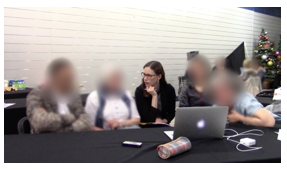
Advisory committee 3—Centre-du-Québec	Decisions on conflicting results. Obtaining feedback on the latest version of the prototype before website programming.	Medium–high-fidelity prototype (version 2). Plenary discussion. Face-to-face and videoconference participation.	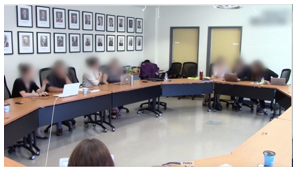

### Emerging Themes

Looking at the results with codes counting only once per document (course-of-action framework of each participant), a total of 5 emerging themes of experience were identified for the entire project: *perception of extrinsic constraints and effects of the situation* (27/74, 36%), *learning together* (14/74, 19%), *frustrating interactions with others* (6/74, 8%), *destabilization* (18/74, 24%), and *getting personal benefits* (9/74, 12%). Table 4 presents the definitions of the themes and the subthemes related to them.

There were important differences depending on the category of participant (caregivers, health and social service professionals, and community workers) and type of session (co-design sessions and AC meetings). *Perception of extrinsic constraints and effects of the situation* was strongly mentioned by health and social service professionals (6/19, 32%) and community workers (16/35, 46%) and less by caregivers (5/20, 25%). It was strong during both types of sessions (co-design sessions: 19/55, 35%; AC meetings: 8/19, 42%). The most important theme mentioned by caregivers was *destabilization* (8/20, 40%), which was also stronger during AC meetings (6/19, 32%) than during co-design sessions (12/55, 22%). *Learning together* was another theme that was strong for health and social service professionals (6/19, 32%). The percentage represents the importance of a theme for a category of participants and the type of session in the overall experience for each.

**Table 4:** Themes and subthemes of participant experience.

Theme	Definition	Subthemes
Perception of extrinsic constraints and effects of the situation	On the basis of the extrinsic constraints related to the situation [22]. Perceptions related to effects of the prescribed tasks (or lack thereof) modulating the course of action of the actor. Can apply to either task during sessions or the organization of sessions.	Impression of a lack of guidance or structure from the moderator Satisfied with useful inputs from the moderator helping them understand Impression of wasting time on circular discussions Expecting to work on a more advanced prototype Perception of insufficient time allowed to reach the objectives Satisfied with the convenience of small groups Wishing they had been able to prepare in advance Satisfied with balanced representation of participant categories (or the opposite) Feeling they are not really participating or not enough Wishing for more facilitation from the moderator to provide a democratic space
Learning together	Inspired by level 3 of the typology of relationships of participation (learning together) by Harder et al [37]. Represents a form of interaction with others where the focus is learning from others’ opinions.	Wanting to help caregivers Wanting to obtain caregivers’ opinion Being able to have access to a diversity of opinions
Frustrating interaction with others	Feelings related to interactions with other participants. Something that a participant (or more) is doing or saying that is annoying to the person. An irritating experience leading to frustration.	Having strong emotions hearing about a caregiver’s situation Annoyed by the confrontation of perspective
Destabilization	Uncomfortable, unbalanced, or disruptive feeling not caused by an interaction with other participants.	Disappointment at the lack of joint efforts on the project Feeling lost, not understanding, or having a lack of knowledge Unsure about which perspective to adopt Perceived incapacity to reach the objective of the task Having trouble with the abstract nature of the task
Getting personal benefits	Positive contribution to personal interests.	Learning about resources Feeling valued by their contribution (caregivers)

### Visual Mapping of Co-occurrences Between Themes and Subthemes

**Figure 3:**
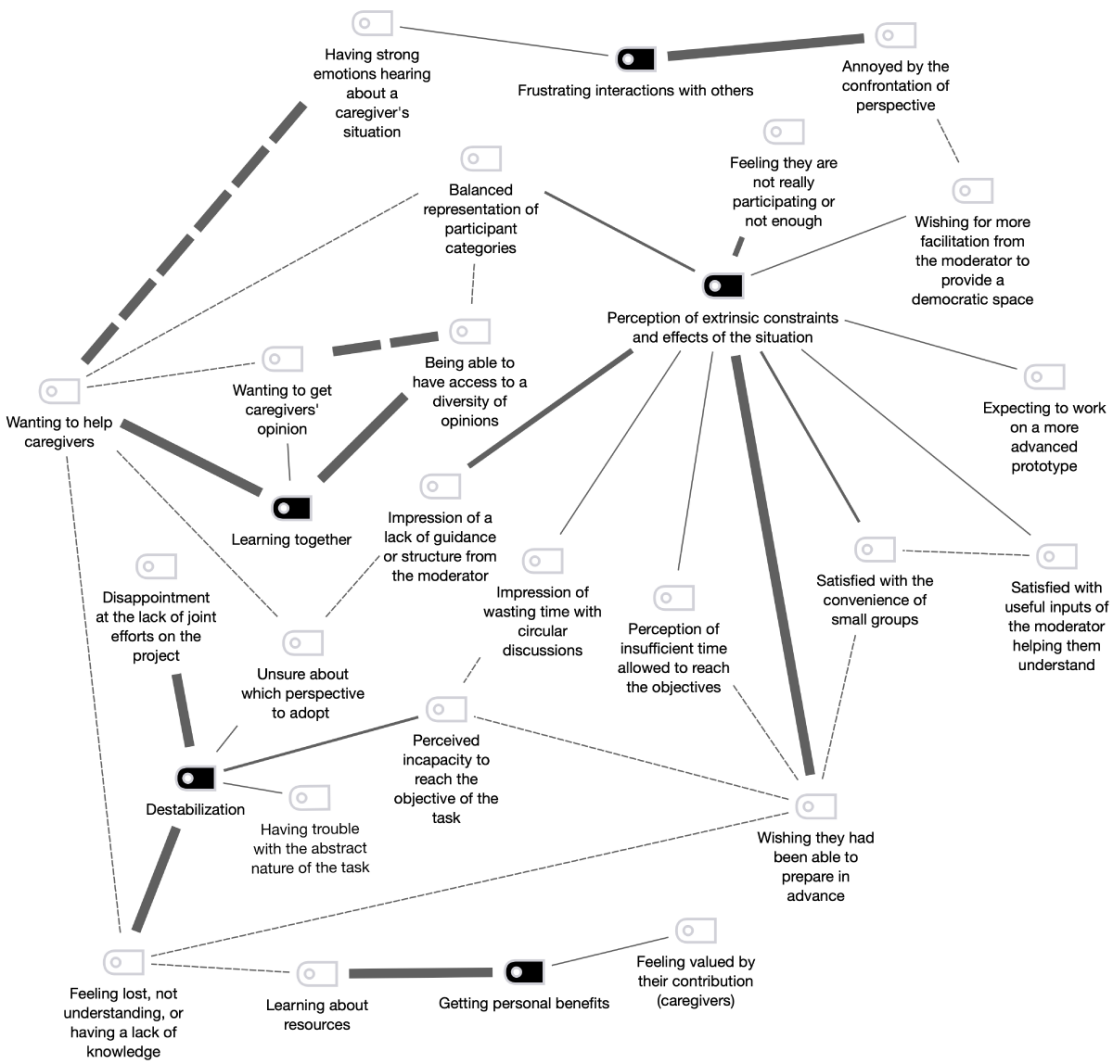
Relationship between mutual themes. This figure was produced using the MAXMap functionality in MAXQDA (VERBI GmbH). Themes are in black, and subthemes are in white connected by solid lines. Dotted lines represent the co-occurrences of codes, and the thickness of the lines represents the importance of the connection (numbers). Adapted from Tremblay [5], which is published under Creative Commons Attribution-NonCommercial-ShareAlike 4.0 International License (CC BY-NC-SA 4.0) [27])

As mentioned in the *Methods* section, the themes were not mutually exclusive. Figure 3 provides a visual representation of the co-occurrences of themes. It also presents a mapping of the experiences of the participants during this co-design research project. The links between themes and subthemes illustrated in this figure indicate the multiple ways in which the experiences of the participants can be understood.

Figure 3 demonstrates the central position of the *perception of extrinsic constraints and effects of the situation* for all sessions, with the prominent star shape and many of its subthemes being linked with other experiences. This theme was strongly mentioned by the participants, and Figure 3 allows us to follow the path of their experience. For example, it shows that *extrinsic constraints* is related to *destabilization* through *impression of a lack of guidance or structure from the moderator*, leading to being *unsure about which perspective to adopt*. The fact that they were *wishing they had been able to prepare in advance*—leading to *feeling lost, not understanding, or having a lack of knowledge*—also connects *extrinsic constraints* and *destabilization*. We can see that the *extrinsic constraints* of *wishing for more facilitation from the moderator to provide a democratic space* led the participants to be *annoyed by the confrontation of perspective,* which was included in *frustrating interactions with others*. More positive effects can also be seen. For example, *extrinsic constraints* leads to *balanced representation of participant categories*, which is linked with *wanting to help caregivers* and *being able to have access to a diversity of opinions*, leading to *learning together*.

**Figure 4:**
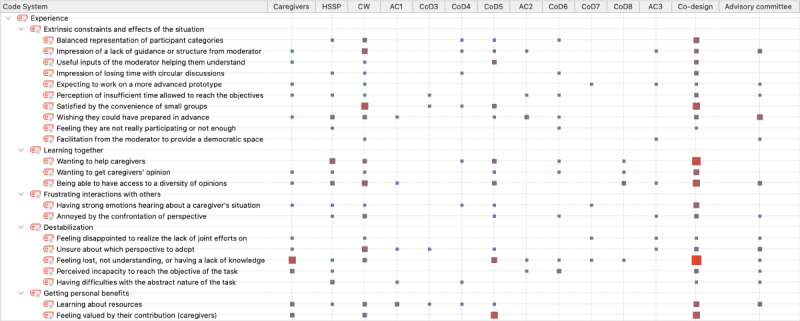
Themes and subthemes by session and type of participant. This figure was produced using the Code Matrix Browser functionality in MAXQDA (VERBI GmbH). The calculation of the square size refers to all the coded segments. AC: advisory committee; CoD: co-design session; CW: community worker; HSSP: health and social service professional.

Figure 4 provides context for the themes and subthemes. This figure offers a situated explanation of Figure 3. The first 3 columns divide the experiences among the 3 categories of participants. The next columns indicate the sessions, and the last 2 columns gather the experience for the type of session (co-design and AC).

### Understanding Experience With the Hexadic Sign

#### Overview

The aforementioned figures provide an in-depth understanding of the experience of the participants if we investigate the signs and their components. The course-of-action methodology produces an important quantity of qualitative data (signs and components). The map helped us select qualitative data to present based on what was significant for the participants, visually highlighted in the map. The sign and its components provide a detailed description of the cognitive aspects underlying the action at the junction of each theme and its subthemes.

#### Preparation and Moderation of Sessions

The map highlights the importance of the role of the research team in the preparation and moderation of the sessions. For example, the link between *wishing they had been able to prepare in advance* and *perceived incapacity to reach the objective of the task* was mentioned by caregiver 11-11 (AC 2) as she did not have sufficient information to properly understand the research project (component R) and would have liked to obtain all the documents before the session (component I). The link between *feeling lost, not understanding, or having a lack of knowledge* and *wishing they had been able to prepare in advance* was also mentioned by caregiver 11-11 at the end of the session (AC 2):

I did not familiarise myself with the document, I only got it today. I realise that it is very focused on computers...I don’t use computers much (U).

The link between *impression of wasting time with circular discussions* and *perceived incapacity to reach the objective of the task* was mentioned by health and social service professional 6-6. The first hour of this session (co-design session 6) was dedicated to presenting the progress of the work, explaining the workshops for this session, and providing information on digital health literacy.

*Destabilization* occurred for 10% (2/20) of the participants during the first and second ACs. Health and social service professional 11-5 was destabilized because she was *having trouble with the abstract nature of the task*. The first AC was held after only 2 co-design sessions, which were mainly focused on user needs. Caregiver 11-11 was destabilized as she did not fully understand her role before engaging in the project. She thought she was just going to share her experience (component A) but realized that the session was putting her back into a work mode (she was a former nurse; component R), which she did not want (component I). She was *feeling lost, not understanding, or having a lack of knowledge* (component S). Although she could have continued to participate as she was part of the AC, she desisted after the session. These 2 reasons were also a source of *destabilization* for other caregivers during co-design sessions (caregiver 5-7, caregiver 6-9, and caregiver 7-7, who was talking about another caregiver participating in the session).

#### Interaction Among Participants

The map and sign also provide a detailed description of the interaction between participants during the sessions, which is a core aspect of the co-design approach. The link between *wanting to help caregivers* and *having strong emotions hearing about a caregiver situation* was mentioned by 10% (2/20) of the participants. The first one was health and social service professional 4-4 (co-design session 4). She realized during the session that the caregiver might be upset at hearing that other caregivers were receiving services, whereas she was not (component I). Health and social service professional 4-4 wanted to help this caregiver during the break (component U) to avoid her going back home discouraged by that (component E). The second participant was community worker 5-1, who was alone with caregiver 5-7 (co-design session 5). He was working for her. She was having a difficult time (component R), and community worker 5-1 felt he needed to help her (component U). This participant was dissatisfied with the lack of *balanced representation of participant categorie*s. He was surprised to be alone with a caregiver (component U) and felt that input from a health and social service professional would have been interesting (component I), explaining the link between this subtheme and *being able to have access to a diversity of opinions*. He mentioned that he would have contributed differently if he had been placed in another group (component I).

The link between *being able to have access to a diversity of opinions* and *wanting to get caregivers’ opinion* was mentioned by 10% (2/20) of the participants. The first one was community worker 5-4 (co-design session 5), who stepped back (component U) as she wanted to let the caregiver talk (component E). She realized that this caregiver was allowing her to obtain another point of view on how caregivers search for resources (component I). The second participant was caregiver 8-9 (co-design session 8), who was with another caregiver in her subgroup workshop. The other caregiver was providing a different opinion from hers, and caregiver 8-9 was interested in seeing how different it was for other caregivers.

The results also show that the caregiving culture was not completely shared by the participants as they were reinterpreting aspects of it. *Frustrating interactions with others* was very strong for health and social service professional 4-4 during co-design session 4. This participant realized during the activity that a caregiver was not receiving the resources she was entitled to (component R). This experience began with a short moment at the beginning of the session (00:37:42). The caregiver said the following:

I have a social worker, but I think we are not high priority for them because I’ve been waiting for 3 months now.

A total of 10 signs from health and social service professional 4-4 were somehow related to this initial situation, with emotions (component U) moving from incomprehension to discouragement and anger. Health and social service professional 4-4 even said during the interview that it was pretty much the end of the session for her:

I would say, at this point, I was not thinking about the tool [anymore]. It pretty much ended my meeting (I).

For community workers, 93% (14/15) of the coded signs were from a single participant (community worker 11-6) during the last AC meeting. Community worker 11-6 had a disagreement with another participant about the language that should be used and the posture behind it (patient-centered).

## Discussion

### Principal Findings

Our objective was to explore the potential of the course-of-action framework [21-23] to describe the intrinsic experience—from the perspective of experience by Dewey [14]—of potential users participating in a co-design research project. Our results showed that this framework was particularly well suited for our objective. *Perception of extrinsic constraints and effects of the situation* appeared to be central in this co-design research project, leading to positive and negative experiences for the participants. The course-of-action framework links the intrinsic description with the extrinsic description. However, the extrinsic constraints and effects of the situation should not be confused with the extrinsic description, which is the description by the researcher performed in the first phase of data collection. The extrinsic constraints and effects of the situation are what the participants identified (their intrinsic description) as elements of the situation that affected their experience. Through their experience, the participants shared the positive and negative effects of the extrinsic constraints of the situation. The results highlight the importance of the role of the research team in preparing and moderating the sessions and provide a detailed description of the interaction between participants during the sessions, which is a core aspect of the co-design approach.

Our results allow us to propose ways to better shape the participant experience [26]. We do not argue that optimizing the experience of the participants will systematically optimize the information obtained by the design. The rationale is that the participation of people is what distinguishes the co-design approach from other design methods. Therefore, they are an essential aspect of data collection. Taking responsibility in the co-design research approach requires reflecting on what designers and researchers can offer participants [38]. This includes offering them a positive and contributory experience to encourage their participation in future co-design projects.

We suggest avenues for shaping the co-design experience as affordances to empower participation [39] reflecting on what was significant in the experience of the participants from their point of view. Affordances, described by Gibson [40], are what the environment provides to the living and, as mentioned by Dewey [14], “[a]t every moment, the living creature is exposed to dangers from its surroundings, and at every moment it must draw upon something in its surroundings to satisfy its needs.” The following affordances are suggestions for co-design researchers to shape the co-design experience for the participants.

### Affordances to Shape the Experience of Co-design

#### Provide Clear Information to the Participants About the Co-design Session in Advance

The participants were *wishing they had been able to prepare in advance*, leading to *feeling lost, not understanding, or having a lack of knowledge,* which in turn affected their participation. Bossen et al [41] noted similar results, identifying project organization as an impediment to user gains. Other negative effects were *feeling they [were] not participating enough* during the long presentation period at the beginning of the session and *expecting to work on a more advanced prototype*, experienced by 10% (2/20) of the participants during the last sessions (co-design sessions 7 and 8). Clear and detailed information provided in advance will allow the participants to know exactly what they will be working on so that they can prepare and have sufficient knowledge to participate and quickly engage in co-design. The participants should be active early in the process, and long, passive presentation periods should be avoided. The information they need could be sent before the session to shorten the introduction part of the process.

#### Work in Small Groups With a Moderator and Ensure a Balanced Representation of Categories of Participants

Among the positive effects, the participants were *satisfied with useful inputs of the moderator helping them understand* and *satisfied with the convenience of small groups*. They were also satisfied with the *balanced representation of participant categories*, with an unbalanced representation leading to negative effects on participant experience. This subtheme was linked with *being able to have access to a diversity of opinions*, which is part of *learning together*. Learning together is indeed a constitutive component of the co-design approach [37]. The small groups and the input of the moderator facilitate the *learning together* experience.

#### Optimize Collaboration by Orienting Toward Positive and Constructive Interactions Among Participants

The participants were *wishing for more facilitation from the moderator to provide a democratic space*, with the lack of facilitation leading some to be *annoyed by the confrontation of perspective*. This is consistent with Tironi [42], who discussed design activities or the process of designing as a space for differences and frictions, reflecting ontological differences among the participants. Dissensus might be a way to innovation [43], but guidance should be provided to avoid transforming dissensus into confrontation, which, in the end, can hinder innovation. We could remind the participants that co-design is a space for dissensus and that all co-designers should adopt a constructive criticism approach. A conflict management protocol could also be helpful.

#### Provide Clear Guidance and Structure

The *impression of a lack of guidance or structure from the moderator* was sometimes related to the participants being *unsure about which perspective to adopt*. This was the case for community workers not knowing whether they should participate in the role of community worker or put themselves in the caregivers’ shoes. This might be specific to this project considering the typical care relationship between service providers and caregivers. Nevertheless, having different categories of participants is inherent to co-design projects. With the goal of adopting empathy in the co-design process [44], it should be clear to the participants which role we want them to adopt for a specific activity.

#### Define Realistic Objectives for the Time Allowed for Each Session

The participants also had a *perception of insufficient time allowed to reach the objectives* and an *impression of wasting time with circular discussions,* both of which pertain to time constraints, also pointed out by Bowen et al [45], leading to *perceived incapacity to reach the objective of the task* in the theme *destabilization*. This might be difficult to achieve as it is not possible to foresee how the participants will engage in the tasks, and some objectives can take longer to achieve than others. The participants need to feel that their contribution was valuable. They ought to be able to have a satisfying experience. Therefore, it should be made clear to them that their contribution is valuable even if not all of the objectives are reached during a session.

#### Allow Participants to Derive Personal Benefit From Their Participation

*Learning together* is part of the benefit that participants can derive from their participation. In this co-design project, other potential benefits for the participants were *learning about resources* and *feeling valued for their contribution*. From an ethical perspective of co-design, we should make a commitment to offer benefits to our participants and assume the responsibility for doing so [38].

#### Guide Participants Toward a Cocreative Design Thinking Mode

Acting as co-designers, the participants are called on to engage in design thinking in terms of designerly ways of knowing [46], which is not necessarily usual for them. In our data, no subthemes or themes were specific to the design of the tools. This appears not to be significant in their experience. Following Manzini [7], we believe that the designers should act as facilitators to help participants understand this mode of thinking and engage in it. It is through this expertise of facilitating design thinking that the designers can offer a meaningful contribution by putting in place the necessary conditions to allow the participants to contribute in their own way.

#### Organize Co-design Projects in Terms of Life Experience and Focus on Empathy Toward the Situation

Co-design research projects should focus on empathy toward the situation and the participants rather than using a solution-oriented approach [42]. We cannot entirely foresee where the project will lead. In this project, the design of the tool was not identified as a theme in the participants’ experience. *Extrinsic constraints and effects of the situation* was the most important theme. More empathy toward the situation might have allowed us to reduce the importance of these constraints, avoid *frustrating interactions with others* by embracing dissensus and orienting co-designers toward constructive interactions, avoid *destabilization* by providing more information in advance, and enhance *learning together*, perhaps being able to reach the last level of participation in the typology by Harder et al [37]: learning as one.

### Contribution of the Course-of-Action Framework to Describe the Experience

The course-of-action methodology required a significant appropriation period and a great deal of time for data collection and analysis, but the results were extremely rich. Using the course-of-action methodology, we were able to gain an in-depth understanding of the cognitive aspects underlying the participants’ experiences. Moreover, the course-of-action framework did not aim to describe the entire session but rather significant parts of it from the participants’ point of view, representing the perspective of experience by Dewey, as pointed out by Laudati and Leleu-Merviel [26]. We believe that the results would not have been as detailed with another methodological approach.

The use of video recording to confront the participants was particularly useful to help them remember the situation and activate their prereflexive consciousness (what they were thinking at that specific moment). Without this, it might have been difficult for the participants to remember exactly what they were thinking. Moreover, we believe that the reconstitution of the sign and its components significantly strengthened and deepened our understanding of the cognitive aspects underlying the actions of the participants (expectations, involvement, referential, and interpretant). We believe that the extra effort of data collection and data analysis was valuable for our objective.

### Challenges and Limitations

Our study had certain limitations. First, the results presented cannot be considered to account for a saturation of the experience for each moment and each step of the co-design project. We had a defined number of data points, and we performed an in-depth analysis of these following the situated cognitive anthropology of the course-of-action framework. The described experience only applies to the participants included in our data collection.

Second, identifying significant moments for the participants was occasionally a challenge. Moreover, significant moments emerged during the self-confrontation interviews, also described by Perrin [47]. Our study shows that, to address this issue, the researcher must remain highly flexible and allow the participant to *guide* the interview by focusing on the moment they want to talk about. Researchers must sometimes temporarily suspend their own involvement in favor of an approach that is open to the participant’s experience [33]. This was especially true as MT had a design background and was sometimes tempted to orient the discussion during interviews to gain more design insights. The partial chronicle helped maintain focus on the session and on the experience of the participant during the interview.

Third, data collection and analysis were influenced by the researchers’ course of action. Although we were not able to find any clear mention of this within the course-of-action scientific community, it is consistent with the epistemological perspective of human activity within the course-of-action framework—the activity is cognitive, situated, and dynamic [48]. These considerations of the activity apply to both participants and researchers. As stated by Leblanc [25], the researcher is not in a passive position with regard to the analyzed situation but, rather, is engaged in a collective program with the participants to understand the activity, seeking a compromise between the scientific community’s rigorous expectations and the expectations of the communities under study. From this perspective, it seems utopian to expect a completely objective analysis. As Theureau [21] said, the researcher is an essential instrument for data collection in anthropological research, simultaneously an observer and an interlocutor. The data are coconstructed through the researcher’s interaction with the participant, and researchers must acknowledge the effects of this interaction on the situations they are studying.

Finally, the results might have been different if the researcher-designer had been completely engaged in facilitating design thinking with the participants during co-design and AC sessions. The researcher-designer was not identified as a designer for data collection purposes. The anonymity of the role of the designer hindered the possibility of completely engaging in a designer-facilitator role. In that sense, we did not completely follow the co-design approach of Manzini [7]. Participants and members of the research team were all co-designers, but we believe that the role of the designer as designer of the experience for participants in the co-design project and designer-facilitator should not be neglected.

### Conclusions

This paper explored what we can learn from participants’ experiences to inform the co-design process. The course-of-action framework strongly contributed to providing a detailed and in-depth description of the experiences of potential users engaging in a co-design research project. We were able to capture what was significant to them from their own perspective. The perception of extrinsic constraints and effects of the situation was the most important theme, leading to positive and negative experiences for the participants. The results highlight the importance of the role of the research team in preparing and moderating the sessions. They also provide a detailed description of the interaction between participants during the sessions. Potential users are essential to the co-design research approach. Researchers and designers should seek to offer them a positive and contributory experience. Reflecting on our results, we proposed affordances to shape the co-design process and, thus, inform researchers and practitioners about potential settings that could lead to a more positive experience for the participants and potentially more fruitful results. Future research should explore how the proposed affordances influence the success of the intervention.

## Abbreviations

AC: advisory committee
